# Gender equality in the Italian academic context. Results from the IGEA project

**DOI:** 10.3389/fpubh.2023.1125496

**Published:** 2023-02-21

**Authors:** Giovanna Deiana, Marco Dettori, Narcisa Muresu, Mariangela Valentina Puci, Laura Saderi, Maria Lucia Piga, Valentina Sias, Daniela Pisu, Maria Antonietta Foddai, Tommaso Gazzolo, Pedro Pablo Fiorini, Lucia Milia, Gavino Mariotti, Giovanni Sotgiu, Antonio Azara, Andrea Piana

**Affiliations:** ^1^Department of Biomedical Sciences, University of Sassari, Sassari, Italy; ^2^University Hospital of Sassari, Sassari, Italy; ^3^Department of Medicine, Surgery and Pharmacy, University of Sassari, Sassari, Italy; ^4^Department of Humanities and Social Sciences, University of Sassari, Sassari, Italy; ^5^Department of Law, University of Sassari, Sassari, Italy

**Keywords:** gender equality, academic staff, university, workplace, occupational stress

## Abstract

The Innovation for Gender Equality in Academia (IGEA) project is focused on the analysis of the gender composition in academia, on the identification of the health needs of the academic population and on the assessment of their organizational wellbeing, in order to promote equal working conditions and opportunities. The study, focused on the identification of health needs, involved the construction of an *ad hoc* questionnaire in order to collect the socio-demographic characteristics and the perception of working environment of the participants. Differences between males and females were evaluated by the Mann-Whitney test, and Pearson Chi-Square or Fisher exact tests as appropriate, highlighting significant differences between genders regarding the occurrence of anxiety, panic, irritation and annoyance related to work activities. A multivariate logistic regression analysis was performed to identify factors associated with the perception of work-related anxiety/panic, showing a direct association with the difficulty in work performance and the work-related stress during the pandemic period, whereas, an indirect association was found with job satisfaction and the feeling of being appreciated by colleagues. Occupational stress can increase the risk of developing physical and mental conditions, also affecting work performance and absenteeism. It is therefore fundamental to plan targeted interventions, implement policies and specific actions, in order to avoid and reduce any differences related to gender.

## 1. Introduction

Italy is among the European countries least committed to gender equality recognition policies. Despite the indications that have been present in national and international regulatory provisions, such as the Lisbon Strategy and the European Research Area Policy Agenda, the achievement of gender equality in the workplace and in the professions still represents an important challenge (1, 2). As a matter of fact, among the 17 Sustainable Development Goals (SDGs) included in the 2030 Agenda for Sustainable Development, signed in 2015 by 193 UN member countries, gender equality (Goal 5) is identified as an objective not yet achieved (3).

Gender, according to the definition of Global Health 50/50, refers to all social aspects that determine roles, positions and relationships among people in a community. Consequently, gender-related characteristics can influence health status and disease susceptibility due to daily activities and lifestyle behaviors, including diet, experiences and perceived stress (4, 5). In particular, psychological stress, which could be a predictor of several somatic and mental disorders, is strongly influenced by gender differences. In-deed, women and men react differently to stress conditions. This is mainly attributable to biological factors, such as hormone release, and their social role which expose them to specific stressors in family and work environments (6).

At the level of workers' health, gender differences have been subjected to careful observation also on the basis of current European and Italian legislation, which introduces the obligation to assess the risk of stress, with particular attention to gender differences (7, 8). In fact, the social and physical work environment, representing a reservoir of multiple physical and psychological work stresses, could have a positive or negative impact on the health status and wellbeing of workers, as well as affecting work performance and productivity. Damage from work stress is well documented and affects virtually all the systems of the human organism, from the cardiovascular system (arterial hypertension) to the endocrine-metabolic system (menstrual disorders, obesity, thyroid diseases), to gastrointestinal disorders (gastritis, ulcer, colitis), mental disorders (depression, neurosis, insomnia, anxiety) and even dermatological disorders such as alopecia, psoriasis, dermatitis (9–12).

The non-specificity and multifactoriality of stress-related pathologies makes it difficult to establish a causal link between these conditions and exposure to work stress. This also explains why the risk of stress has long been neglected in medicine and prevention in the workplace. While, on the one hand, nowadays there is increased sensitivity toward the issue, on the other hand, observing the signs of the tangible presence of a condition influenced by gender inequalities is difficult and requires a holistic approach between different disciplines. In particular, as regards the aspects directly affecting state of health, even before highlighting a compromised situation including states of full-blown disease, it would be advisable to resort to preliminary observations that allow the identification of risk conditions for health attributable to differences in gender (13–17).

The gender gap is a particularly persistent phenomenon in the academic world (18, 19). In this regard, it is important to understand the genesis of inequalities in the phases of an academic career and in roles in order to study the dynamics through which asymmetries are an obstacle to equality, also considering health needs and work-related stress, and to propose the genre as a knowledge and programming tool. Understanding and addressing the factors that produce gender imbalances, therefore, has a positive impact not only in terms of equality of opportunity, but also of general efficiency and excellence (20–22).

In order to anticipate any compromised state of health, it would therefore be useful to operate through a monitoring system aimed at identifying the risk factors or predisposition to the event, known and recognized by the scientific literature.

The primary aim of the present study was to assess the health needs of academic staff, in terms of health status, workplace condition, relationship, and psychological aspects, with particular focus on gender differences. Secondly, we evaluated potential factors associated with the perception of work-related anxiety and panic. These findings could represent essential points to plan targeted interventions aimed at reducing health inequalities in the academic setting.

## 2. Materials and methods

### 2.1. Study setting

The IGEA project, with reference to the mythological Goddess of health, is focused on the analysis of the gender composition of the University of Sassari, as a starting point for gender budgeting and gender auditing activities, in order to equip the University with such a tool as the Gender Report, to monitor the performance plan in terms of gender equal opportunities.

The University of Sassari is a medium-sized Italian University, with a population of about 13,500 students and 1,095 employees. With its 10 Departments and over 650 teachers from various Universities throughout Italy, the University of Sassari offers face-to-face and distance learning in both the humanities and the sciences. The University has over 40 interdisciplinary research centers and 12 libraries, presenting a wide choice for medical practice, and boasts cooperation relationships with ~500 Universities participating in the Erasmus program.

This project is divided into four phases. In the first phase, the gender context of the University was analyzed, in order to investigate its gender composition, providing a snapshot of the gender distribution in all levels of academic staff. In the second phase, we analyzed the gender regulatory framework of the University. The third phase involved the construction and administration of a questionnaire to identify the health needs of the academic population and assess their organizational wellbeing, in order to promote equal working conditions and opportunities. The last phase involved the drafting of the University's Gender Budget.

In particular, we focused our attention on the identification of the health needs of the academic population, in order to underline the existing situation and anticipate any health problems of employees attributable to a subsistent condition of inequality based on gender.

### 2.2. Questionnaire construction and administration

The study involved the construction of an *ad hoc* questionnaire, containing numerous clinical-health and economic-organizational indicators linked to the accessibility, the environment and the relationship established with colleagues and managers of the work structures in order to evaluate the main problems present and, consequently, identify any necessary corrective actions.

The questionnaire consists of a first section collecting the socio-demographic characteristics of the participants, followed by 24 questions (Q1–Q24) related to the participants' perception of their working environment. The questions, reproduced below in the results section (see the tables in the Results section), are divided into five sections: perception about health status (Q1–Q5), perception about workplace (Q6–Q12), psychological aspects (Q13–Q17), perception about workplace interactions and relationship (Q18–Q21) and perception about pandemic scenario (Q22–Q24). The definition and subsequent evaluation of the individual question were programmed through a balanced scale of 6 values, where 1 corresponds to completely disagree and 6 to completely agree.

The questionnaire was drawn up using a Google form and was previously tested in a pilot study by a sample of University employees (data not published) in order to evaluate the questionnaire's comprehensibility. The questionnaire was administered during the post-pandemic period, in 2022, by email and newsletters, to the 1,095 employees of the University of Sassari, composed of professors, researchers, language experts and technical-administrative staff.

### 2.3. Statistical analysis

Participants' characteristics were described using medians and 25–75° percentiles (IQR) or absolute and relative (percentages) frequencies. Shapiro-Wilk test was used to assess the normality of quantitative data. Differences between males and females were evaluated by the Mann-Whitney test, and Pearson Chi-Square or Fisher exact tests as appropriate. Multivariate logistic regression analysis was performed to identify factors associated with the perception of work-related anxiety/panic (Q13). This outcome was classified as follows: low level (grouping score values of 1, 2, and 3), and high level of anxiety/panic (grouping score values of 4, 5, and 6). The results were reported as Odds Ratios (ORs) and corresponding 95% Confidence Intervals (95% CI). Candidate variables were selected based on clinical and statistical significance (at univariate analysis). Two-tailed *p* < 0.05 was considered statistically significant. Data analyses were carried out with STATA 17 (StatsCorp, Texas, USA).

## 3. Results

### 3.1. Socio-demographic and occupational characteristics of the participants

A total of 205 subjects (19% response rate) completed the survey entirely: 61.1% were female, sample median (IQR) age was 54 (46–59) years with significant differences between females and males [median (IQR) 51 (44–58) years and 57 (46–61) years, respectively] (*p* = 0.02) (Table 1). Over half of participants had a partner (54.2%) and only one quarter (25.1%) were single. The highest percentage of respondents were affiliated to scientific areas (42.7%), followed by administrative (31.2%) and humanistic (26.1%) ones. Participants were mainly rep-resented by full and associate professors (53.2%), followed by technicians and administrative workers (45.8%).

**Table 1:** Participants' socio-demographic characteristics.

**Variables**		**Study cohort**	**Females**	**Males**	***p*-value**
		**(No. 205)**	**(No. 124; 61.1%)**	**(No. 79; 38.9%)**	
Median (IQR) age, year		54 (46–59)	51 (44–58)	57 (46–61)	0.02
Marital status	Single	51 (25.1)	34 (27.4)	17 (21.5)	0.17
	Married	110 (54.2)	62 (50.0)	48 (60.8)	
	Cohabiting	15 (7.4)	11 (8.9)	4 (5.1)	
	Divorced	16 (7.9)	12 (9.7)	4 (5.1)	
	Separated	9 (4.4)	3 (2.4)	6 (7.6)	
	Widowed	2 (1.0)	2 (1.6)	0 (0.0)	
Setting, *n* (%)	Administration	62 (31.2)	43 (35.5)	18 (23.7)	0.15
	Sciences	85 (42.7)	51 (42.2)	34 (44.7)	
	Humanities	52 (26.1)	27 (22.3)	24 (31.6)	
Personnel, *n* (%)	Mother tongue linguistic experts	2 (1.0)	2 (1.6)	0 (0.0)	0.14
	Technical/Administrative/Library staff	93 (45.8)	62 (50.0)	31 (39.2)	
	Teaching staff	108 (53.2)	60 (48.4)	48 (60.8)	
Educational level, *n* (%)	Middle-school diploma	3 (1.5)	2 (1.6)	1 (1.3)	0.25
	High-school diploma	26 (12.8)	13 (10.5)	13 (16.5)	
	Three-year degree	3 (1.5)	0 (0.0)	3 (3.8)	
	Undergraduate degree	27 (13.3)	19 (15.3)	8 (10.1)	
	Specialist degree (5-year)	27 (13.3)	19 (15.3)	8 (10.1)	
	PhD	87 (42.9)	53 (42.7)	34 (43.0)	
	Other	30 (14.8)	18 (14.5)	12 (15.2)	

### 3.2. Participants' perception of their working environment

Results as median (IQR) scores for each question were reported in Table 2.

**Table 2:** Median and IQR values for the 24 questions.

**Perception of working environment (score 1–6)**	**Study cohort (no. 205)**	**Females (no. 124)**	**Males (no. 79)**	***p*-value**
	**Median (IQR)**			
**Perception about health status**				
Q1. Are you satisfied with your state of health?	5 (4–5)	5 (4–5)	5 (4–5)	0.37
Q2. How informed do you feel you are on health issues?	4 (4–5)	4 (4–5)	5 (4–5)	0.69
Q3. Are you satisfied with your diet?	4 (4–5)	4 (4–5)	5 (4–5)	0.27
Q4. Are you satisfied with your level of physical exercise?	3 (3–4)	3 (2–4)	4 (3–4)	0.19
Q5. Are you able to enjoy your hobbies in your free-time?	4 (3–5)	4 (3–5)	4 (3–5)	0.06
**Perception about workplace**				
Q6. Do you feel that you have adequate space for your work requirements?	4 (3–5)	4 (3–5)	4 (3–5)	0.05
Q7. Do you use video terminals when performing your duties?	6 (5–6)	6 (5–6)	6 (5–6)	0.54
Q8. Do you find your workplace comfortable?	4 (3–5)	4 (3–5)	4 (3–5)	0.05
Q9. Can you perform your work at a sustainable pace?	4 (3–5)	4 (3–5)	4 (3–5)	0.52
Q10. Do you have the opportunity to take sufficient breaks?	4 (3–5)	4 (3–5)	4 (3–5)	0.16
Q11. Do you make use of the recreational spaces in your workplace?	2 (1–4)	2 (1–3)	2 (1–4)	0.05
Q12. Are you informed about activities and events organized by your administration?	4 (3–5)	4 (3–5)	4 (4–5)	0.02
**Psychological aspects**				
Q13. In relation to your work, do you experience states of anxiety, panic or feeling down, depressed?	3 (2–4)	3 (2–5)	2 (1–4)	0.002
Q14. In relation to your work, do you feel irritable, nervous or angry?	3 (2–4)	3 (2–4)	2 (1–4)	0.004
Q15. Are you satisfied with your job?	4 (4–5)	4 (3–5)	5 (4–5)	0.006
Q16. Do you feel optimistic about your professional future?	3 (2–4)	3 (2–4)	4 (2–5)	0.01
Q17. Do you make use of psychological support and stress management services?	1 (1–1)	1 (1–1)	1 (1–1)	0.32
**Perception about workplace interactions and relationship**				
Q18. Do you feel you can count on the support/help of the people you work with?	4 (3–5)	4 (3–5)	5 (4–5)	0.42
Q19. Do you consider your manager a reference point?	4 (3–5)	4 (3–5)	4 (3–6)	0.13
Q20. Do you feel that you are respected or appreciated by the people you work with?	5 (4–5)	5 (4–5)	5 (5–5)	0.0003
Q21. With respect to your work, do you feel that you belong to a group or community?	4 (3–5)	4 (3–5)	5 (4–5)	0.0002
**Perception about pandemic scenario**				
Q22. Do you think your work-related stress level has increased during the pandemic?	3 (2–5)	3 (2–5)	3 (1–4)	0.19
Q23. Do you feel that the pandemic has made it more tiring to perform your job?	4 (3–5)	4 (3–5)	4 (3–5)	0.93
Q24. Do you feel that the pandemic has negatively influenced social relations in your workplace?	3 (2–4)	3 (2–4)	3 (1–4)	0.05

Considering the questions relative to the perception of health status (Q1–Q5), participants declared feeling high satisfaction regarding personal health status and lifestyle habits, with a median score ranging from 4 to 5. The analysis of the results relating to the perception of workplace (Q6–Q12) showed that workers were sufficiently satisfied with working space [median (IQR) score 4 (3–5)] and workload [median (IQR) score 4 (3–5)]. Moreover, participants considered their place of work comfortable, with a median (IQR) score of 4 (3–5). Whereas, a lower median (IQR) score [2 (1–4)] was observed regarding the use of recreational spaces and/or services at work.

Although a poor level of anxiety/panic (Q13) was reported in items related to psychological aspects [median (IQR) score: 3 (2–4)], a significant difference between females and males was registered [median (IQR) score: 3 (2–5) and 2 (1–4), respectively, *p* = 0.002]. Similar results were observed considering the occurrence of irritation and annoyance (Q14). Significant differences were found in work satisfaction (Q15), with a lower score in women than in men in reference to their current work [4 (3–5) and 5 (4–5), respectively, *p* = 0.006], and also considering their vision about their professional future (Q16) [3 (2–4) and 4 (2–5), respectively, *p* = 0.01].

Regarding perception about workplace interactions and relationships, similar results between genders were observed considering the support offered by teamwork, the relationship with the manager, and the feeling of being appreciated by colleagues (Q18–Q20). However, a significant difference was observed concerning the feeling of belonging to a group (Q21) in females, who showed lower median scores than males [4 (3–5) and 5 (4–5), respectively, *p* = 0.0002].

The questions relating to the perception of the pandemic scenario did not show differences by gender, in terms of difficulty in work performance, work-related stress, and quality of social relations in the workplace (Q22–Q24).

**Figure 1:**
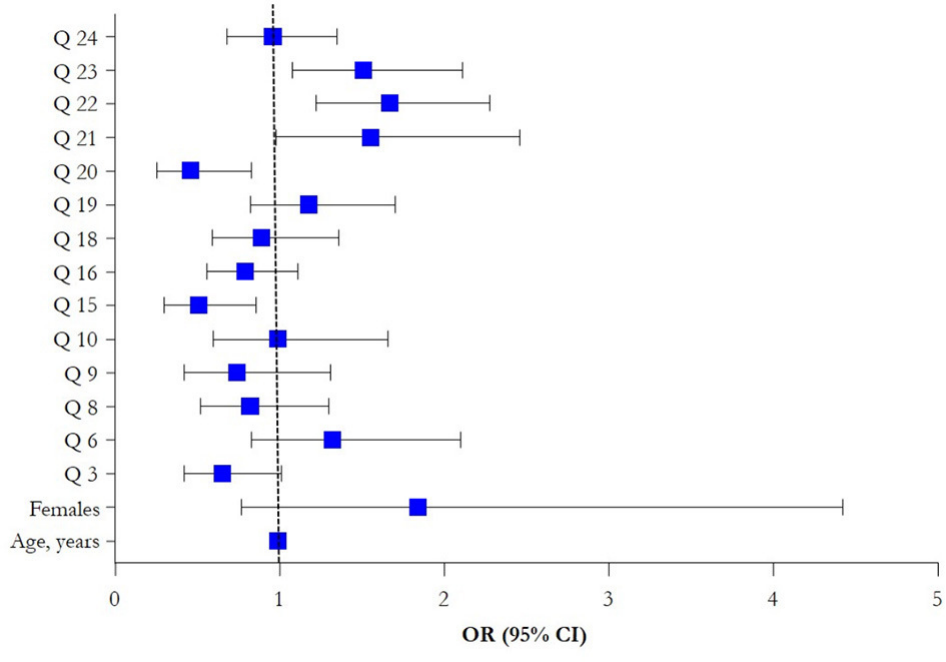
Forest plot of multivariate logistic regression results (OR, 95% CI).

The multivariate analysis (Table 3, Figure 1) showed that the difficulty in work performance [OR (95% CI): 1.51 (1.08–2.11), *p* = 0.02], and work-related stress [OR (95% CI): 1.67 (1.22-2.28), *p* = 0.001] during the pandemic period were associated with an increased probability of work-related anxiety/panic (Q22, Q23). Whereas job satisfaction [OR (95% CI): 0.51 (0.30–0.86), *p* = 0.01], and the feeling of being appreciated by colleagues [OR (95% CI): 0.46 (0.25–0.83), *p* = 0.001] decreased the likelihood of work-related anxiety/panic (Q15, Q20).

**Table 3:** Logistic regression to assess relationship between socio-demographic characteristic and perception of working environment, and work-related anxiety/panic.

**Variables**	**Univariate analysis**		**Multivariate analysis**	
	**OR (95%CI)**	***p*** **-value**	**OR (95%CI)**	***p*** **-value**
*Age, years*	0.98 (0.95–1.01)	0.10	0.99 (0.95–1.04)	0.77
*Females*	2.20 (1.20–4.04)	0.01	1.84 (0.77–4.42)	0.17
*Teaching staff*	1.38 (0.78–2.42)	0.27	–	-
**Perception about health status**				
Q1. Are you satisfied with your state of health?	0.80 (0.60–1.07)	0.14	–	-
Q2. How informed do you feel you are on health issues?	0.96 (0.74–1.24)	0.75	–	-
Q3. Are you satisfied with your diet?	0.60 (0.44–0.81)	0.001	0.65 (0.42–1.01)	0.06
Q4. Are you satisfied with your level of physical exercise?	0.83 (0.68–1.02)	0.08	–	-
Q5. Are you able to enjoy your hobbies in your free-time?	0.83 (0.67–1.01)	0.07	–	-
**Perception about workplace**				
Q6. Do you feel that you have adequate space for your work requirements?	0.74 (0.60–0.91)	0.004	1.32 (0.83–2.10)	0.23
Q7. Do you use video terminals when performing your duties?	0.95 (0.69–1.29)	0.73	–	-
Q8. Do you find your workplace comfortable?	0.76 (0.62–0.93)	0.009	0.82 (0.52–1.30)	0.41
Q9. Can you perform your work at a sustainable pace?	0.53 (0.40–0.69)	< 0.0001	0.74 (0.42–1.31)	0.30
Q10. Do you have the opportunity to take sufficient breaks?	0.65 (0.51–0.82)	< 0.0001	0.99 (0.60–1.66)	0.99
Q11. Do you make use of the recreational spaces in your workplace?	0.80 (0.66–0.97)	0.03	–	-
Q12. Are you informed about activities and events organized by your administration?	0.82 (0.67–1.01)	0.06	–	-
**Psychological aspects**				
Q15. Are you satisfied with your job?	0.46 (0.34–0.61)	< 0.0001	0.51 (0.30–0.86)	**0.01**
Q16. Do you feel optimistic about your professional future?	0.57 (0.46–0.70)	< 0.0001	0.79 (0.56–1.11)	0.18
**Perception about workplace interactions and relationships**				
Q18. Do you feel you can count on the support/help of the people you work with?	0.66 (0.53–0.82)	< 0.0001	0.89 (0.59–1.36)	0.60
Q19. Do you consider your manager a reference point?	0.72 (0.60–0.87)	0.001	1.18 (0.82–1.70)	0.38
Q20. Do you feel that you are respected or appreciated by the people you work with?	0.46 (0.33–0.63)	< 0.0001	0.46 (0.25–0.83)	0.01
Q21. With respect to your work, do you feel that you belong to a group or community?	0.69 (0.55–0.85)	< 0.0001	1.55 (0.98–2.46)	0.06
**Perception about pandemic scenario**				
Q22. Do you think your work-related stress level has increased during the pandemic?	1.83 (1.49–2.24)	< 0.0001	1.67 (1.22–2.28)	0.001
Q23. Do you feel that the pandemic has made it more tiring to perform your job?	1.59 (1.29–1.96)	< 0.0001	1.51 (1.08–2.11)	0.02
Q24. Do you feel that the pandemic has negatively influenced social relations in your workplace?	1.43 (1.18–1.73)	< 0.0001	0.96 (0.68–1.35)	0.81

## 4. Discussion

Gender inequality and workplace disparities in academia are a multi-faceted issue which encompasses economic discrepancy, dissimilar career opportunities, and unequal representation in leadership positions. There is extensive evidence of gender imbalances in academia, for which a plethora of possible explanations have been proposed, from differences in family responsibilities to resource allocation, collaboration, role stereotypes, academic course, and working climate (23–27). This disparity is greatest in positions of leadership, suggesting that women must choose between career advancement and their personal life (28). Consequently, women reported a lower sense of belonging and forging relationships within the workplace, which certainly does not facilitate their integration and advancement (29, 30). As highlighted by the British Council, the higher education, including academy, should represent an incubator of innovation for future society. Nevertheless, the education system sometimes promotes gender inequality and discriminatory norms for women. The same report suggests several objectives to contrast gender inequalities, as increased awareness among workers, equal access to resources and opportunity, collaboration, legal support for workers and changes in attitudes and practices (31).

Our survey aimed to identify the potential risk factors for the health status of academic workers, with particular attention to gender differences. Overall, the results made it possible to detect a general satisfaction among personnel with regard to their health status and lifestyle habits, revealing that both males and females are sufficiently or often satisfied as regards their health perception, although it is possible to highlight certain critical issues for which it would be necessary to implement specific policies and actions.

Specifically, as regards the perception about workplace, despite the overall satisfaction with regard to space and comfort in the workplace, it is necessary to investigate the condition of dissatisfied people in more detail, hopefully by carrying out an accurate survey on the distribution of spaces and of staff within the University structures. Moreover, the impossibility, declared by many interviewees, to take advantage of the recreational spaces present in their workplace is worrying. In this case, it is necessary to investigate further to understand whether what has been highlighted is linked to an excessive workload, which would not allow sufficient breaks to be taken during the working day, or to the actual lack, in some working environments, of adequate recreational spaces.

With reference to the perception about workplace interactions and relationships, it could be useful to investigate in depth the diminished feeling of belonging to a community of a number of staff and the lack of trust in their manager, as highlighted by some studies even in pre-pandemic periods and in different working contexts (32). Finally, with regard to the psychological aspects, it is necessary to be able to intercept personnel who are unable to adequately manage their workload and above all those who show states of anxiety or panic and who feel irritable or nervous in relation to their work. In this case, it could be useful to provide for the establishment of a listening desk capable of providing psychological support and/or the scheduling of interviews managed by multidisciplinary teams that specifically deal with workplace wellbeing (33).

It is also clear, from a general point of view, how the pandemic situation has made work more onerous, making it more tiring to carry out one's work, sometimes increasing stress levels and negatively affecting social relationships. Differently from our expectations, we did not find significant differences in the impact of COVID-19 between males and females, probably linked to the narrow sample size of the study. However, numerous studies reported that the pandemic had a greater impact on women, particularly in working mothers who dealt with growing housework and childcare, the long-term effect of which could increase the gender gap over the next few years (34). Based on the current literature, it is important to emphasize how the COVID-19 pandemic may have affected work relationships. The low perception of being part of a group, particularly present in women, may have been influenced also by the social restriction imposed during the pandemic period. However, these hypotheses require further evaluation to be confirmed (35).

Significant differences were found between genders regarding the occurrence of anxiety, panic, irritation and annoyance related to work activities. As a matter of fact, occupational stress can increase the risk of developing physical and mental conditions, such as cardiovascular diseases, burnout, insomnia and depression (36, 37). Moreover, stress conditions could affect work performance and could be linked to absenteeism from work. On this basis, it appears essential to understand the reasons and plan interventions to prevent and address these challenges. Onset of work-related stress may have different causes in males and females, mainly related to biological, physiological and social factors (38–40). Our findings are consistent with previously published data that define excessive workload and the university workload model as an “anxiety machine”, attributable to various causes, such as precarious contracts, unattainable expectations, career advancement difficulties and excessive use of controls and metrics (41).

Various studies evidenced how lack of promotion and barriers in the career ladder represent one of the major sources of stress in females. Moreover, the perception of career barriers has been linked to negative health consequences and reduced individual gratification, also affecting productivity, innovation, commitment, and leadership (42–45). According to previous studies, we found lower satisfaction in women, probably related to difficulties in relationships with superiors and colleagues and also to the negative perception of future professional positions. As a matter of fact, job security and relationships with team members have beneficial effects on life and work satisfaction, particularly among women (46). These aspects are particularly alarming in the academic workplace, where, despite the level of education being similar between genders, women are underrepresented in leadership and senior positions (47, 48).

### 4.1. Study limitations

This study should be interpreted in view of some limitations mainly due to its design (i.e., cross-sectional survey), where information and answers were self-reported, to the small amount of complete questionnaires analyzed and to the low response rate. As such, this study likely represents only the views of a convenience sample of academic staff in North Sardinia. Nevertheless, the results above could be useful for determining priority health problems, planning targeted interventions and implementing the University's policies and actions, as the assessment of health needs can become an opportunity to deepen, as shown by various studies and research on the subject, the broader theme of wellbeing and quality of life at work. Moreover, this finding suggests that we must rephrase the discussion about gender inequality around the sustainability of woman's careers in academia, with significant administrative and policy implications (49, 50).

## 5. Conclusions

This study reported the health needs and wellbeing status of academic workers, including professors and administrative staff. Our results showed several differences in emotional stress response between females and males. We found that women showed a higher level of anxiety and greater concern about future prospective than men. Moreover, work performance and stress related to the pandemic period were considered risk factors for workplace stress and anxiety, whereas job satisfaction and collaborative teamwork had a positive effect on workers wellbeing.

Our findings support that Italian academia needs to reduce the gender gap and to improve scientific careers and advancement for women. Moreover, more actions aimed to establish good teamwork should be evaluated and pursued. Therefore, it is our intention to carry out the present survey routinely, to monitor the improvement of gender equality over time, as well as extend this survey to other University institutions and compare the data with other national and international contexts.

## Data availability statement

The raw data supporting the conclusions of this article will be made available by the authors, without undue reservation.

## Author contributions

All authors made a significant contribution to the work starting from the conception, study design, execution, acquisition of data, analysis, interpretation, in drafting, revising, or critically reviewing the manuscript, gave final approval of the version to be published, have agreed on the journal to which the article has been submitted, and agree to be accountable for all aspects of the work.
